# Facile and Simple Post Treatment Ball Milling Strategy for the Production of Low-Cost TiO_2_ Composites with Enhanced Photocatalytic Performance and Applicability to Construction Materials

**DOI:** 10.3390/ma16144931

**Published:** 2023-07-10

**Authors:** Kabuyaya Kighuta, Sun-Woo Kim, Yao-Long Hou, Kwang-Pill Lee, Wha-Jung Kim

**Affiliations:** 1 Department of Civil Engineering, Kyungpook National University, Daegu 41566, Republic of Korea; dankabuyaya@knu.ac.kr; 2 GOONWORLD Corporate Research Institute, Dong-gu Inovalley 26 Road 9-115, Daegu 41065, Republic of Korea; kplee@knu.ac.kr; 3 Department of Chemistry Education, Chosun University, Gwangju 61452, Republic of Korea; swookim@chosun.ac.kr; 4 College of Railway Engineering, Zhengzhou Railway Vocational and Technical College, Zhengzhou 451460, China; hylmm8988@hotmail.com

**Keywords:** commercial TiO_2_, composites, ball milling, NO_x_ photodegradation, efficiency comparison, eco-friendly construction materials

## Abstract

A facile and cost-effective approach assisted by ball milling (BM) of commercial titanium dioxide (TiO_2_), has been utilized to develop cheaper and efficient construction materials. At least three of the commercial and cheaper TiO_2_ samples (BA01-01, BA01-01+ and R996, designated as A1, A4 and R1, respectively) were selected and subjected to BM treatment to enhance their photocatalytic efficiencies, if possible. It was noted, that the samples A1, A4 and R1 were typical composites of TiO_2_ and calcium carbonate (CaCO_3_) and contained varying proportions of anatase, and rutile phases of TiO_2_ and CaCO_3_. Two of the highly efficient commercial TiO_2_ samples, Degussa P25 (simply designated as P25) and ST01 (Ishihara Ind.) were selected for making benchmark comparisons of photocatalytic efficiencies. The BM treated TiO_2_ samples (designated as TiO_2_-BM with respect to A1, A4 and R1) were evaluated for photocatalytic efficiencies both in both aqueous (methylene blue (MB)) and gaseous (NO_x_) photodegradation reactions. Based on detailed comparative investigations, it was observed that A1-BM photocatalyst exhibited superior photocatalytic performances over A4-BM and R1-BM, towards both MB and NO_x_ photodegradation reactions. The difference of NO_x_ photodegradation efficiency between the mortar mixed with A1-BM and that mixed with ST01, and P-25 at 15% were 16.6%, and 32.4%, respectively. Even though the mortar mixed with A1-BM at 15% composition exhibited a slightly lower NO_x_ photodegradation efficiency as compared to mortar mixed with the expensive ST01 and P-25 photocatalysts, the present work promises an economic application in the eco-friendly construction materials for air purification considering the far lower cost of A1. The reasons for the superior performance of A1-BM were deduced through characterization of optical properties, surface characteristics, phase composition, morphology, microstructure and particle size distribution between pristine and BM treated A1 using characterization techniques such as diffuse reflectance spectroscopy, X-ray photoelectron spectroscopy, X-ray diffraction analysis, field emission scanning electron microscopy and particle size analysis.

## 1. Introduction

The extensive research developments with semiconductor photocatalysts lead to the conclusion that photocatalysis is the most efficient, sustainable and convenient remediation method for treating the majority of pollutants [1,2,3]. Titanium dioxide (TiO_2_) is prevalently used as the photocatalyst due to its multi-faceted functional properties, that include high oxidation efficiency, non-toxicity, high photo stability, chemical inertness and environmental friendliness. The evolved photocatalytic oxidation technology based on TiO_2_ could be effectively employed to degrade various pollutants and the chief advantage is that it can thoroughly mineralize organic matter to water, CO_2_, and nontoxic components, thereby avoiding secondary pollution. Amongst the commercial TiO_2_, P-25 (Evonik, Germany) has been widely used as a reference photocatalyst showing superior performances in photocatalysis for various gaseous pollutants [4,5]. However, P-25 is relatively very expensive for practical large-scale usage and hence considering the cost, several other commercial TiO_2_ have been manufactured. However, almost all cheaper commercial TiO_2_ materials show far inferior photocatalytic performances as compared to P-25 and their practical applications are limited. It is highly demanding to develop cheaper TiO_2_ with photocatalytic performances that are closer to P-25 but this could be attempted through modification of the cheaper commercial TiO_2_ and by employing adequate but less expensive procedures.

Based on the large number of publications related to the photocatalytic applications of TiO_2_, it has been inferred that a number of factors could influence the efficiency of the photocatalytic performances of pristine TiO_2_ photocatalysts. Those factors could be broadly categorized into (i) intrinsic properties of the photocatalyst (TiO_2_) and (ii) external reaction conditions. To enhance the photocatalytic performance of Pristine TiO_2_, a large number of efficient strategies have been evolved that include crystalline phase tuning [6,7], density of lattice and surface defects [8,9], particle size alteration [10,11] and porous modifications [12]. As regards crystalline phases, TiO_2_ exists in three main phases: anatase, brookite and rutile [13]. TiO_2_ containing both anatase and rutile phased crystallites, showed much enhanced photoactivity as compared to single-phase titania. Typically, P-25, a de facto standard used to understand photocatalysis is a mixed-phase titania photocatalyst showing superior performance over single phase commercial photocatalysts [14]. TiO_2_ nanoparticles with a size of 25 nm showed superior photoactivities to nanoparticles with a size of 15 nm [15]. A number of operational parameters such as concentration of the pollutant, amount of photocatalyst loading, light intensity used, and pH of the medium influence the photocatalytic activity of TiO_2_ [16]. Modification strategies of TiO_2_, both physical and chemical routes, have been reviewed [17,18].

Ball milling (BM) is a simple mechanical technique that can be conveniently used to create deformations in the crystal structure, generation of metastable phases and surface modifications of TiO_2_ [19]. A significant 130-fold enhancement of the photocatalytic properties of TiO_2_ was noticed for BM treated photocatalyst and such an enhancement was explained due to the disordered, amorphous state, together with the srilankite phase formation upon milling [20]. Of note, BM is an eco-friendly, cost-effective and a mass production feasible technique which can be applied both under dry and wet conditions. The inducement in the phase transformation from anatase to rutile with production of material having a higher photocatalytic activity in UV light has been demonstrated [21]. Recently, we have successfully demonstrated 90-fold enhancement in the photocatalytic activity for a commercial TiO_2_ using sequential BM and acid treatment [22].

In this work, we selected three relatively cheaper commercial TiO_2_ samples, namely, BA01-01, BA01-01+ and R996 (the prices per kg are presented in Table 1), designated as A1, A4 and R1, respectively, and post-modified through BM treatment for improving the photocatalytic properties. The BM treated samples were tested for photocatalytic efficiencies both in aqueous and gaseous photocatalytic reactions. The aqueous methylene blue (MB) photodegradation and gaseous NO_x_ photodegradation studies were independently performed with the BM treated TiO_2_ samples and the photodegradation efficiencies were compared with the proven efficient P-25 and ST-01 photocatalysts. It is important to note that the majority of earlier research works on the production of cheaper and efficient TiO_2_ were constrained by two issues; (i) use of complicated and costlier in situ TiO_2_ modification procedures and (ii) inadequate conclusions deduced by evaluating either gaseous or aqueous photodegradation efficiencies. The present work employs a simple post treatment (BM) strategy on a few commercially known cheaper TiO_2_ samples in order to achieve photocatalytic performance comparable to the expensive P-25 and ST-01. Knowing the extensive use of TiO_2_ as a promising additive to building/construction materials such as cement pastes, mortars and concretes for incorporating functional properties to construction materials, this work focused on developing cheaper and more efficient materials for construction purposes. We evaluated BM treated TiO_2_ as a cheaper additive for developing construction materials as our strategy allows large scale applicability in the construction industry.

## 2. Materials and Methods

### 2.1. Materials

Various pristine photocatalysts were purchased from China (A1, A4, R1), Germany (P25), and Japan (ST01). The composition, price, and company of origin of the pristine photocatalysts are summarized in Table 1. The aqueous solution of MB was purchased from SAMCHUN Company Ltd., Seoul, South Korea and utilized without any additional treatment. The characterization details (CD), in terms of instrument and company (manufacturer), for X-ray diffraction (XRD), X-ray photoelectron spectroscopy (XPS), diffuse reflectance spectroscopy (DRS), scanning electron microscopy (SEM), particle size analysis (PSA) and BM treatment, are detailed as supporting information (please refer to SI-CD).

### 2.2. Preparation of BM Treated Samples

The photocatalysts A1, A4, and R1 were subjected to BM treatment for 3 h to determine their photocatalytic activity characteristics. The powdery photocatalyst and the ceramic ball were put into the AV-1 ball miller at a ratio of 1 to 10. The ball mill rotating stand AV-1 manufactured by AS ONE Corporation in Japan was used to prepare A1-BM, A4-BM, and R1-BM photocatalysts for 3 h at 650 rpm. Distilled water was added to the mixture as well. Afterwards, the obtained photocatalytic paste was dried on a hot plate stirrer at about 100 °C. Prior to any photodegradation experiment, the prepared powdery photocatalysts were cooled down in the laboratory environment.

The plain mortar samples were manufactured utilizing standard sand, ordinary Portland cement having a density of 3.15g/cm^3^, and water according to KS L 5201 (ISO 679:2009 [23]) regulations. The photocatalytic mortar samples were prepared in two different ways. Firstly, the photocatalyst powder was mixed with the mortar at various ratios. Secondly the plain mortar samples were coated with the TiO_2_ photocatalysts. About 0.5 g of photocatalyst powder and 1 g of water were mixed and directly applied to the surface of the mortar plate samples. The proportion of the mixture is presented in Table 2.

**Figure 1:**
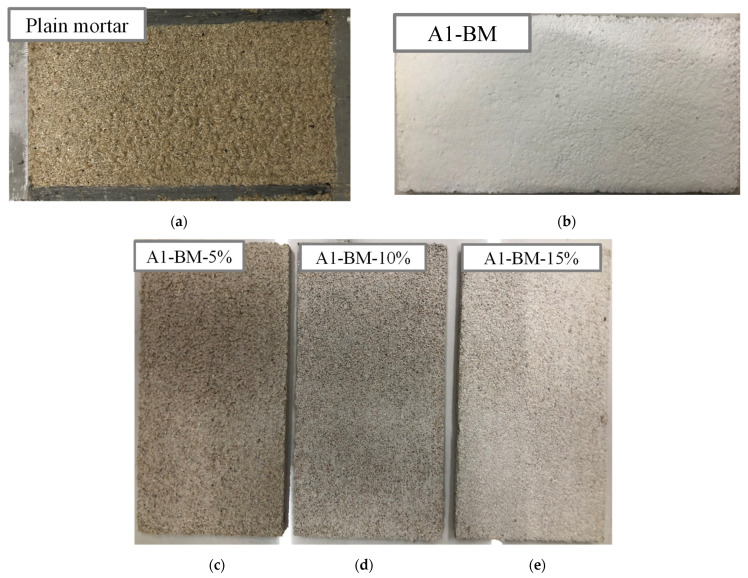
Typical mortar plate samples: (**a**) Plain mortar in mold, (**b**) Mortar sample coated with A1-BM, (**c**) Mortar mixed with A1-BM-5%, (**d**) A1-BM-10% and (**e**) A1-BM-15% photocatalyst.

The water to cement (w/c) ratio, and the sand quantity were kept constant for all the mortar samples. The mortar was mixed in accordance with ASTM C1329-05 [24] regulations. Powdery dry materials such as sand, cement and photocatalyst were first mixed homogeneously. Approximately 70% of the water was poured and mixed; the remaining 30% of water was added and mixed as well. The mortar sample size was 100 mm long, 50 mm wide and 5 mm high. After pouring the mortar into the mold, compaction of mortar was carried out to reduce the air voids in the mortar. The molded samples were covered with a polyethylene sheet for preventing water evaporation during the curing for 24 h in the laboratory environment. Then, the samples were removed from the mold and cured at room temperature (23 °C) for 28 days. Prior to the photocatalytic experiment, all the prepared mortar thin plate samples were dried for 1 h at low temperature (100 °C). Figure 1 presents the snapshots of the typical manufactured mortar plate samples.

### 2.3. Photocatalytic Experiment Program 

#### 2.3.1. Photodegradation of MB (Aqueous)

Typically, the photocatalytic performance of the activated TiO_2_ photocatalyst (fabricated as described in Section 2.1) was investigated by taking decomposition of aqueous MB as the model reaction studied under UV light irradiation (UV lamp (20 W, λ = 352 nm), Sankyo Denki Co., Ltd., Tokyo, Japan). Prior to irradiation, 0.1g of activated TiO_2_ photocatalyst powder was added to the aqueous solution of MB (10 mg/L) and stirred well for 10 min. The photocatalytic experiment was performed for a total volume of MB of 200 mL. After stirring, the dark photocatalyst including MB solution was kept in a dark chamber for 1 h to hasten the adsorption–desorption equilibrium. Subsequently, the MB solution containing the photocatalyst was irradiated under UV-light, and stirred constantly, while keeping the temperature at 23 °C. At periodic time intervals, aliquots were withdrawn successively from the bulk MB solution and the photocatalyst (TiO_2_ particles) was removed from the MB solution by filtration. The concentration of MB after each stage of photodegradation was assessed by recording UV–Vis spectra (UV–Vis spectrophotometer S-3100 (SCINCO Co., Ltd., Seoul, Republic of Korea) of the filtered MB solution. The concentration changes in MB were determined by monitoring the absorbance of MB at 665 nm. The photodegradation experimental setup is presented in Supplementary Materials Figure S1.

#### 2.3.2. Photodegradation of NO_x_

**Figure 2:**
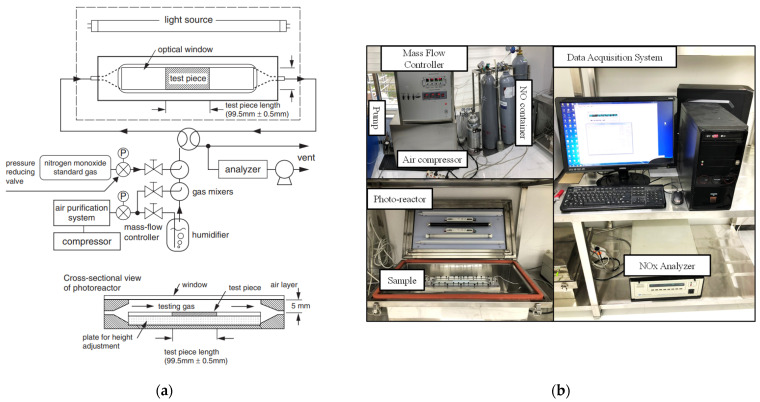
(**a**) Schematic illustration of NO_x_ removal test (JIS R 1701-1:2004) (**b**) Experimental setup.

NO_x_ removal experiments were carried out according to the JIS R 1701-1:2004 standard [25]. The photoactivity of the samples was measured through the photoreactor placed in a stainless box whose dimensions were 620 mm × 430 mm × 285 mm. The photoreactor was 430 mm long, 100 mm wide and 40 mm high. On the top of the cover of the stainless box were installed light sources to induce the photoactivity as illustrated in Figure 2.

The size of the sample in the photoreactor was 100 mm long, 50 mm wide and 5 mm high. The UV-lamp emitting UV rays between 310~400 nm was used to illuminate the photocatalytic mortar sample surface at the intensity of 1000 µW/cm^2^ for 3 h. The distance from the top surface of the mortar thin plate sample to the optical window of the photoreactor was about 10 mm. The prepared mortar sample was introduced to the photoreactor, then the mass flow controller was used to adjust the flow rate of the NO gas, water vapor, and air. The concentrations of NO_x_, NO, and NO_2_ were simultaneously recorded by the chemiluminescence NO_x_ analyzer-Model 200E. However, this work focused and presented only the NO_x_ concentration variation curves. The photocatalytic experiment was carried out when NO_x_ was stabilized at 1000 ppb for 30 min after reaching the adsorption–desorption equilibrium in dark conditions. The UV-light was turned off and the stabilized NO_x_ concentration was monitored for various time intervals. A typical NO_x_ removal scheme is depicted in Figure S2. Figure 2a,b presents the schematic illustration of the NO_x_ removal test and experimental setup. NO_x_ removal performance was computed using the following equation:

(1) $${\mathrm{NO}}_{x}\left(\%\right)=\frac{{\mathrm{NO}}_{x}{}_{\mathrm{in}}-{\mathrm{NO}}_{x}{}_{\mathrm{out}}}{{\mathrm{NO}}_{x}{}_{\mathrm{in}}}\times 100$$

where ${\mathrm{NO}}_{x}{}_{\mathrm{in}}$ represents the initial concentration of NO_x_, and ${\mathrm{NO}}_{x}{}_{\mathrm{out}}$ the recorded concentration at the end of the photodegradation process.

## 3. Results and Discussion

### 3.1. MB Photocatalytic Degradation

**Figure 3:**
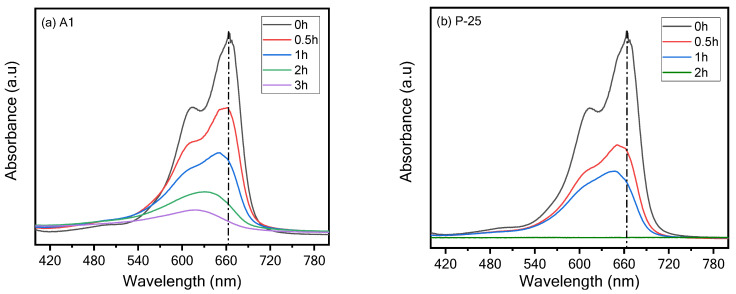

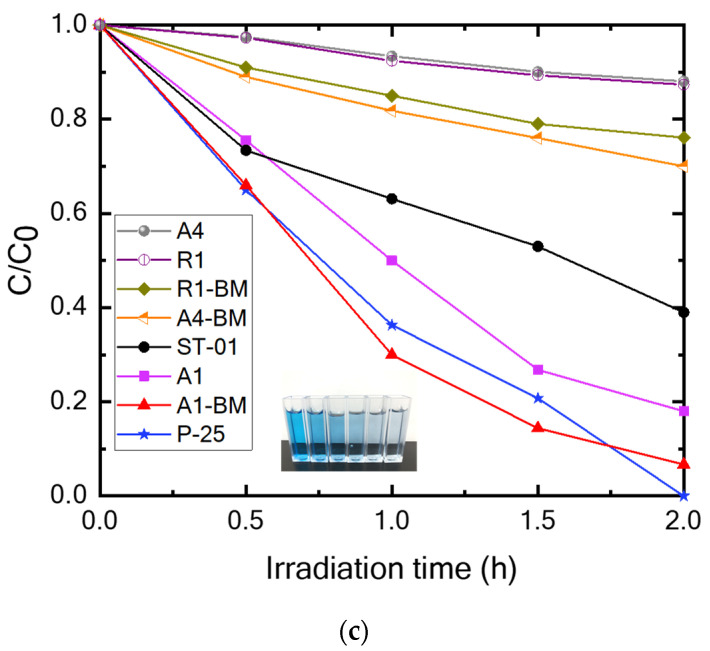
Typical UV–Vis absorption spectra of photodegradation of MB dye using (**a**) A1, (**b**) P-25, (**c**) photodegradation of MB dye under UV-light.

The photocatalytic activity of the photocatalysts (Table 1) was investigated by recording the gradual concentration decrement of MB in presence of each powdery photocatalyst scattered in the solution under UV-light irradiation. Figure 3 presents the absorbance spectra of the photodegradation of MB solution by the previously mentioned TiO_2_ photocatalysts (Table 1). Typical absorbance spectra recorded for A1 and P-25 photocatalysts are displayed in Figure 3a,b.

The computed photodegradation efficiencies for the various samples are displayed in Figure 3c. The absorbance peak decrements can be noticed after a period of 0.5 up to 3 h under UV irradiation. A4 and R4 samples (Table 3) revealed the slowest photocatalytic degradation because MB was not decomposed significantly for 3 h.

The P-25 sample (Figure 3b) presented the fastest photocatalytic decomposition for MB because 100% photodegradation was noticed within 2 h. The A1-BM sample (Figure 3c) presented significant photodegradation of MB compared to other expensive TiO_2_ photocatalysts (except for the P-25 sample as 100% photodegradation was noticed) within 3 h due to BM.

While the samples designated as A1, A4 and R1 comprised of composites of CaCO_3_ with varying proportions (Table 1), A1 has the highest 57.4% CaCO_3_, the other TiO_2_ samples (STO1 and P-25) present varying anatase and rutile compositions. Sample A1 is the cheapest amongst the selected TiO_2_ samples (Table 1).

Table 4 shows the pseudo-first order kinetic parameters of MB photodegradation by the samples listed in Table 1.

**Figure 4:**
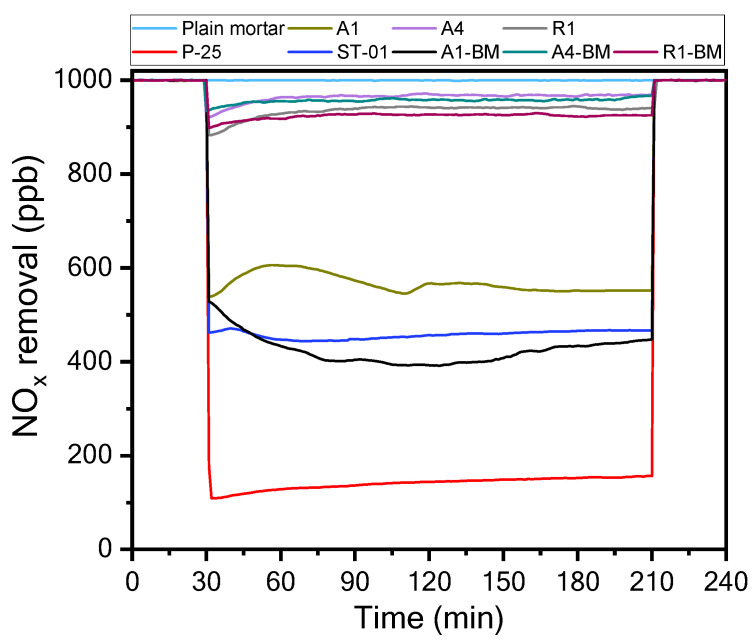
NO_x_ removal of plain mortar, TiO_2_ and BM treated photocatalysts.

Samples P-25 and STO1 exhibited higher photodegradation rate constants for MB photodegradation as compared to pristine A1, A4 and R1 samples, as inferred from the slopes presented inside the plots in Figure 4. A1-BM, A4-BM and R1-BM samples showed increased rate constants as compared to A1, A4 and R1 samples signifying that BM treatment influences the rates of MB photodegradation. Typically, the A1-BM sample has a rate constant (1.38 s^−1^) nearer to or even higher than the P-25 (1.06 s^−1^) sample and far greater than the rate constant of STO1 (0.442 s^−1^), which implies that A1 can be tuned to be an affective photocatalyst by using BM treatment.

### 3.2. Photodegradation of NO_x_

#### 3.2.1. Mortar Specimens Coated with Photocatalysts

Figure 4 compares the photocatalytic NO_x_ removal concentration of the low-cost post-treated A1-BM-coated mortar plate and the mortar samples coated with the expensive photocatalysts (Table 1).

The photodegradation experiments were performed under UV-light (Figure 2). NO_x_ concentration vs. time curves showed a variation in NO_x_ concentration for all the photocatalyst-coated mortar plates over a period of 210 min. The plain mortar was utilized as a reference sample. The average of NO_x_ concentrations after the photodegradation process after 210 min were 999.8, 566.7, 963.7, 955.2, 934.9, 923.2, 140.9, 457.7, and 424.1 ppb for the plain mortar, A1, A4, A4-BM, R1, R1-BM, P-25, ST-01, and A1-BM-coated mortar samples, respectively.

The results showed that A1-BM, A4-BM and R1-BM samples presented an increment in NO_x_ photodegradation compared to the non-treated samples A1, A4, and R1. Amongst the BM-treated samples, A1-BM exhibited a significant increment in NO_x_ photodegradation compared to the mortar sample coated with the expensive photocatalyst ST01. This inferred that the mortar sample coated with the A1-BM photocatalyst presented high photocatalytic activity due to BM treatment. The highest NO_x_ photodegradation was exhibited by the P-25 coated mortar sample. However, P-25/ST01 coated mortars used very expensive photocatalysts and their practicality in construction materials is not promising. Therefore, with a simple BM treatment, the low cost A1-BM photocatalyst is well suited for its practicality as it presented higher NO_x_ photodegradation as compared to the expensive photocatalyst ST01 and nearly comparable efficiencies to P-25.

**Figure 5:**
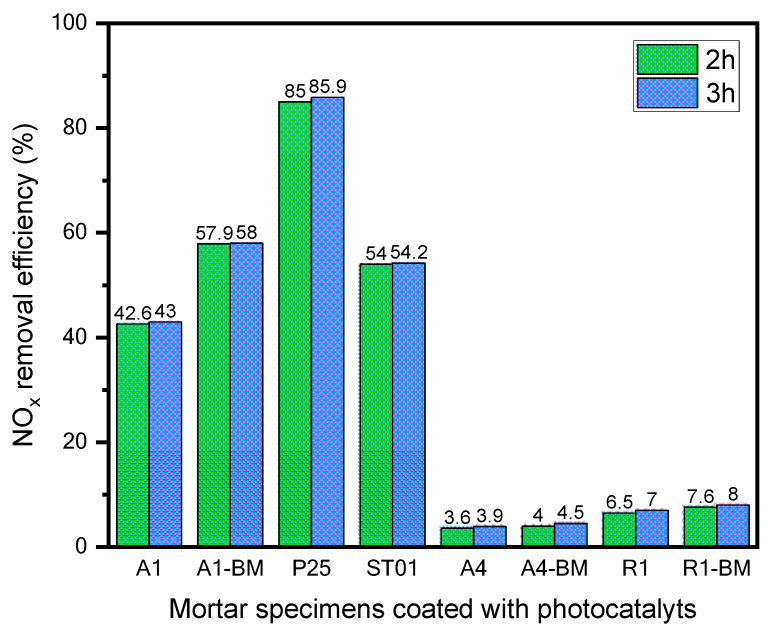
Photodegradation efficiency of NO_x_.

Figure 5 compares the photodegradation efficiency of NO_x_ for mortar samples coated with TiO_2_ photocatalysts during a period of 2 and 3 h.

It can be noticed that NO_x_ photodegradation efficiency over 2 h is very similar to that of 3 h for all the mortar samples coated with TiO_2_ photocatalysts. Therefore, the photodegradation efficiency of NO_x_ is discussed only over 3 h in the next section. This may be due to the saturation of photocatalytic sites for reaction at the surface by 2 h of irradiation.

The results showed that the mortar samples coated with pristine photocatalysts A1, A4, R1 exhibited an NO_x_ photodegradation efficiencies of 43%, 3.9%, and 7%, respectively. Whereas, the mortar samples coated with BM-treated photocatalysts, A1-BM, A4-BM, and R1-BM presented NO_x_ photodegradation efficiencies of 58%, 4.5%, and 8%, respectively. One can infer that the mortar sample coated with A1-BM photocatalyst presented a significant photodegradation efficiency of NO_x_ as compared to mortar samples coated with pristine and other BM-treated photocatalysts. In addition, NO_x_ photodegradation efficiency of the mortar sample coated with A1-BM photocatalyst was slightly higher than that of ST01-coated mortar sample, with a difference of 3.8%. The difference of NO_x_ photodegradation efficiency between A1-BM and P-25-coated mortar samples was 27.9%. It can be concluded that the mortar sample coated with A1-BM photocatalyst promises excellent NO_x_ photodegradation activity due to simple and low-cost BM treatment. This confirmed the economic applicability of the A1-BM photocatalyst to building and construction materials.

#### 3.2.2. Mortar Mixed with the Photocatalysts

**Figure 6:**
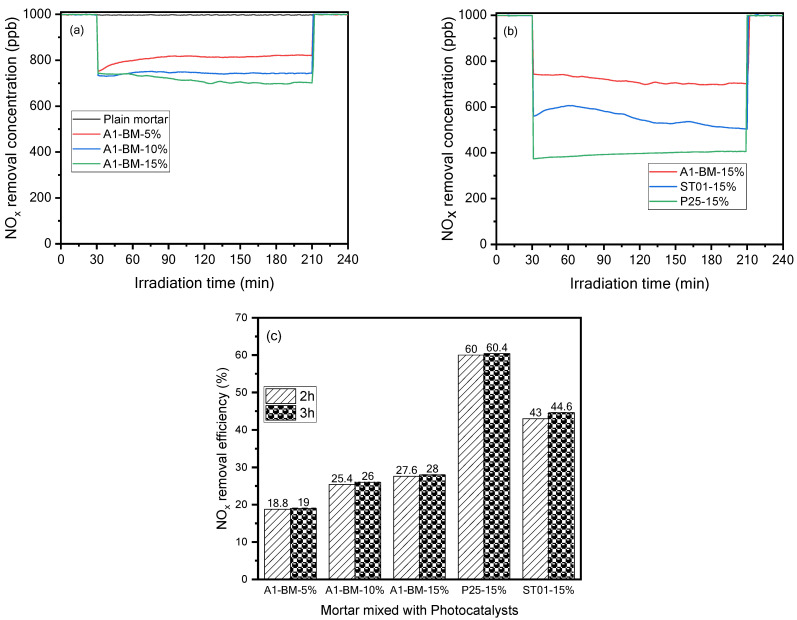
(**a**,**b**) NO_x_ Photodegradation of mortar mixed with the photocatalysts (**c**) NO_x_ removal efficiency of mortar mixed with photocatalysts.

In order to develop the eco-friendly construction material, the photocatalyst A1-BM was selected due to its promising economic use and significant NO_x_ photodegradation as mentioned in the previous section. It was mixed at various ratios (5%, 10%, and 15%) with the mortar, as shown in Table 2. The plain mortar was considered the reference sample. The average of NO_x_ concentrations after the NO_x_ photodegradation process during 210 min of the mortar samples being mixed with the A1-BM photocatalyst at 5%, 10%, and 15% inclusions in mortar were 806.9, 745.7, and 723.6 ppb, respectively, with an initial 1000 ppb NO_x_ concentration. The results showed that NO_x_ removal increased with the increase in the A1-BM photocatalyst amount as presented in Figure 6a.

The mortar mixed with A1-BM photocatalyst at 15% exhibited higher NO_x_ photodegradation. This might have been due to the particle amount of A1-BM photocatalyst submerged beneath the surface of the mortar during the compaction of the mortar sample during the manufacturing process. The number of particles of A1-BM photocatalyst submerged beneath the surface of the mortar mixed at 15% might have been higher as compared to the particle number of A1-MB on the surface of the mortar mixed with 5%, and 10%. Figure 6b compares NO_x_ removal concentration variations during 210 min of the mortar being mixed with A1-BM, ST01, and P-25 photocatalysts at 15%. The NO_x_ concentrations remaining after photodegradation were 723.6, 570.2, and 395.5 ppb for the mortar samples mixed with A1-BM, ST01, and P-25 photocatalysts at 15%, respectively. Figure 6c presents the bar chart showing the difference in NO_x_ photodegradation efficiency between the mortar mixed with A1-BM, ST01, and P-25-photocatalysts. The mortar sample that showed the lowest NO_x_ photodegradation efficiency of 19% was the sample mixed with A1-BM at 5%; whereas the mortar mixed with P-25 at 15% exhibited the highest NO_x_ photodegradation efficiency of 60.4% compared to that of mortar samples mixed with ST01 (44.6% at 15% loading) (Figure 6c). The difference in NO_x_ photodegradation efficiency between the mortar mixed with A1-BM and that mixed with ST01, and P-25 at 15%, were 16.6%, and 32.4%, respectively. Although the mortar mixed with A1-BM at 15% exhibited lower NO_x_ photodegradation efficiency compared to mortar mixed with the expensive ST01 and P-25 photocatalysts, it still promises to be an economic application for eco-friendly construction materials for air purification.

Knowing that A1-BM exhibited superior NO_x_ photodegradation over A4-BM and R1-BM photocatalysts and comparable photodegradation efficiencies with the expensive P-25 and ST01, the following sections explore the reasons behind this.

### 3.3. Optical Property Analysis

**Figure 7:**
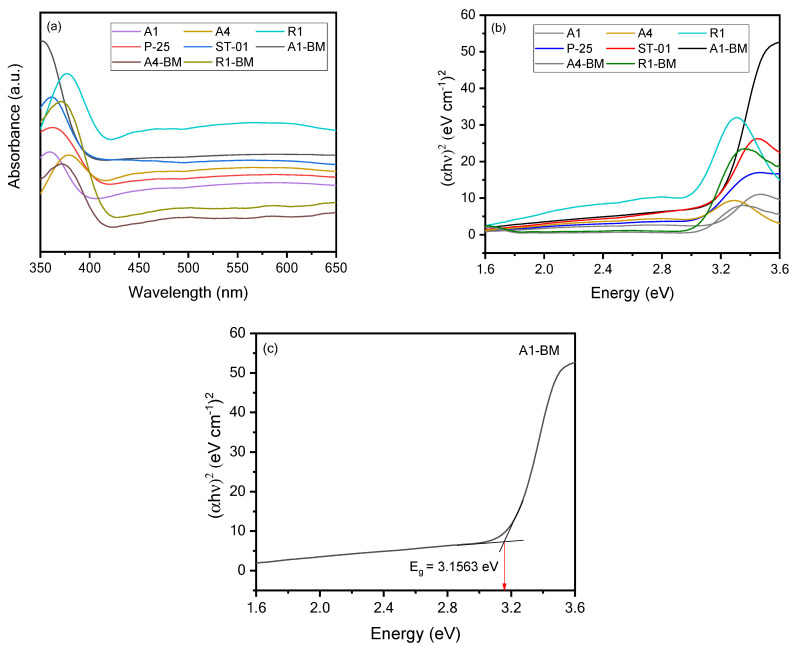
(**a**) UV-DRS patterns, (**b**) Tauc plots of TiO_2_ and BM treated photocatalysts and (**c**) Typical Tauc plot of A1-BM.

Figure 7 presents the comparison between the diffuse reflectance (DR) UV–Vis spectra of the various TiO_2_ samples listed in Table 1.

All the DRS spectra exhibit a typical single absorption edge in the range 380–390 nm, that can be assigned to the band-to-band transition in titania (anatase/rutile). Figure 7a informs that the absorption spectra of the A1, A4 and R1 and their BM treated samples are nearly identical, inferring that their optical band gaps could be the same. The optical band gap (E_g_) of the TiO_2_ samples was evaluated from UV–Vis DR spectral data. The E_g_ of the TiO_2_ samples was determined from the (αhν)^2^ against photon energy (hν) plots (Figure 7b) using the Tauc equation: αhν = (hν − E)^n^, where, n = 1/2 for direct band gap, and 2 for indirect band gap and h, m, a, and E_g_ stand for the Planck’s constant, frequency, absorption coefficient, and the band gap energy, respectively. The direct allowed model (Figure 7b) fits well to this TiO_2_ band structure and is consistent enough to compare amongst all the samples. The E_g_ value of the samples were deduced through the intersection point obtained from the extrapolation (αhν)^2^ against photon energy (hν) plots (Figure 7c). The values of E_g_ of the samples deduced for all the samples are listed in Table S3. On perusal, it is inferred that there is no significant influence of the amount of CaCO_3_ or BM treatment on the E_g_ of TiO_2_ samples.

### 3.4. XRD Analysis

**Figure 8:**
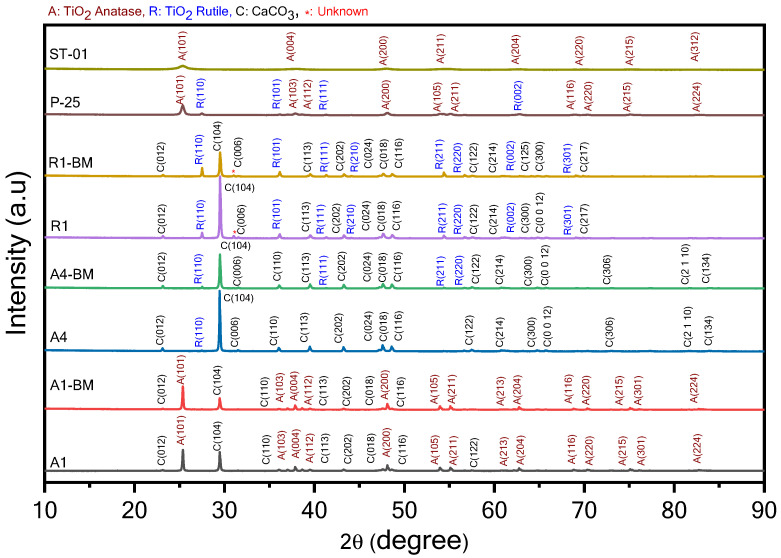
XRD patterns of the photocatalysts.

Figure 8 presents the XRD pattern of A1, A1-BM, A4, A4-BM, R1, R-BM samples that contain CaCO_3_, with varying proportions along with TiO_2_ (Table 1) and the pure P-25 and STO1 TiO_2_ samples.

The XRD patterns of A1, A4 and R1 samples showed the predominant diffraction peaks at 2θ = 23.1, 29.4°, 31.4°, 36.0°, 39.4°, 43.2°, 47.5°, 48.5°, 56.6 a° and 57.4°, which refer to its (012), (104), (110), (113), (202), (024), (018), (116), (211) and (112) facets, respectively (JCPDS PDF2 standard card 05–0586), and confirm that the CaCO_3_ crystals exist in the trigonal calcite phase and the respective crystal planes. While the XRD pattern of A4 and R1 shows the presence of few rutile diffraction peaks (2θ = 27.48 (110), 54.33 (211) etc.), A1 predominantly shows anatase phase diffraction peaks (JCPDS card no. 21-1272). The XRD pattern of A1, A1-BM, A4, A4-BM, R1 and R-BM shows a slight shift in peak positions and changes in intensities that inform the probable disturbance in the crystal phase of TiO_2_. Based on these details, it can be inferred that the crystal phase parameters are altered by BM treatment. Additionally, the BM treatment of A1 causes a small decrement in crystallite size (Table 5). A more detailed analysis was performed by calculating the lattice parameters of the crystal phase in the samples (Table 5).

The calcite phase in sample A1 was rhombohedral with lattice parameters of a = 4.976 Ǻ and c = 16.982 Ǻ, which is in accordance with JCPDS: 96-600-9668. The lattice parameter a in A1 does not change by BM treatment as in A1-BM the lattice parameter in the x-direction, the value of a, is closer to the A1 sample. However, the lattice parameter c is significantly shifted from 16.982 for the A1 sample to 17.025 for the A1-BM sample, meaning that BM treatment elongated the C-axis. The unit cell volume of trigonal crystal is increased from 373.1 for A1 to 421.05 A1-BM, signifying the unit cell expansion, which may be due to the probable modification in the crystal phase by doping. Considering the isoelectronic nature of Ca^2+^ and Ti^4+^ and the ionic radius of these ions, doping is probable. The crystallite sizes of calcite decreased from A1 to A1-BM (Table 5), which can be ascribed to the defects that occurred in the transformation process during BM treatment. The rutile phase lattice parameters in A4 and R1 are not altered much by BM treatment. Therefore, it can be inferred that the binding of CaCO_3_ and TiO_2_ particles in A4-BM and R1-BM is expected to occur at the interfacial region of the particles, rather than any binding through a chemical or physical nature. The absence of any peaks at 2θ = 22.6 and 32.8 means there is a negligibility of phase transformation from stable calcite to meta stable versions during BM treatment for A1, A4 and R1 samples.

### 3.5. SEM Analysis

**Figure 9:**
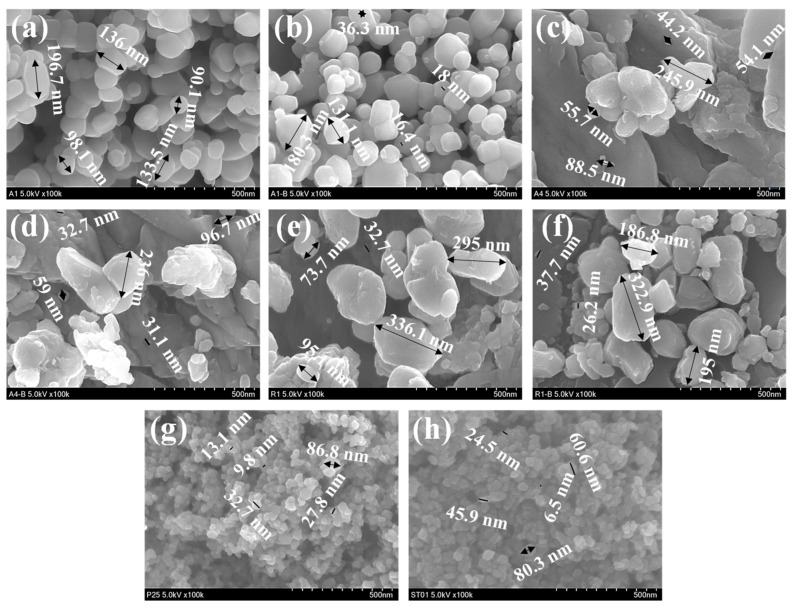
SEM images of (**a**) A1 (**b**) A1-BM (**c**) A4 (**d**) A4-BM (**e**) R1 (**f**) R1-BM (**g**) P25 (**h**) ST01.

Figure 9 depicts the morphology of A1, A1-BM, A4, A4-BM, R1, R1-BM, P-25 and STO1 samples.

The images in Figure 9 of A1, A4 and R1 inform that particles mostly exist in a rhombohedral shape, which is the predominant morphology of calcite. The surface of the grain structure is mostly smooth arising from particle stuffing [26]. The SEM images of A1-BM, A4-BM and R1-BM also retain the rhombohedral shapes, with variation in dimensions as indicated in Figure 9. From the calculation results, the average grain size in the sample is 3 nm, which means that a micron structure is formed in CaCO_3_. Bonds between grains tend to agglomerate and are not evenly distributed [27]. This can occur as a result of the synthesis process which does not allow the aggregation process to occur, as is the case with the nanostructure synthesis method [28]. Based on the literature, the existence of spherical and rhombohedral shapes of CaCO_3_ crystals correspond to vaterite and calcite, respectively [29]. The negligible proportion of spherical particles in SEM images of A1-BM, A4-BM and R1-BM informs that vaterite transformation did not occur due to BM treatment. The SEM images of P-25 and ST01 show a spherical shape with lesser agglomeration of nanoparticles and this may be due to absence of aggregation of primary TiO_2_ particles due to conditions maintained in the crystal’s growth during preparation [30]. The average sizes of the particles of pristine and BM treated samples are presented in Table S4. While BM treatment causes a significant decrease in particle size of the A1 sample (from 136 nm to 76 nm), a marginal decrease was witnessed for A4 and R1 samples. (Table S4).

### 3.6. XPS Analysis

#### 3.6.1. XPS Survey Analysis

**Figure 10:**
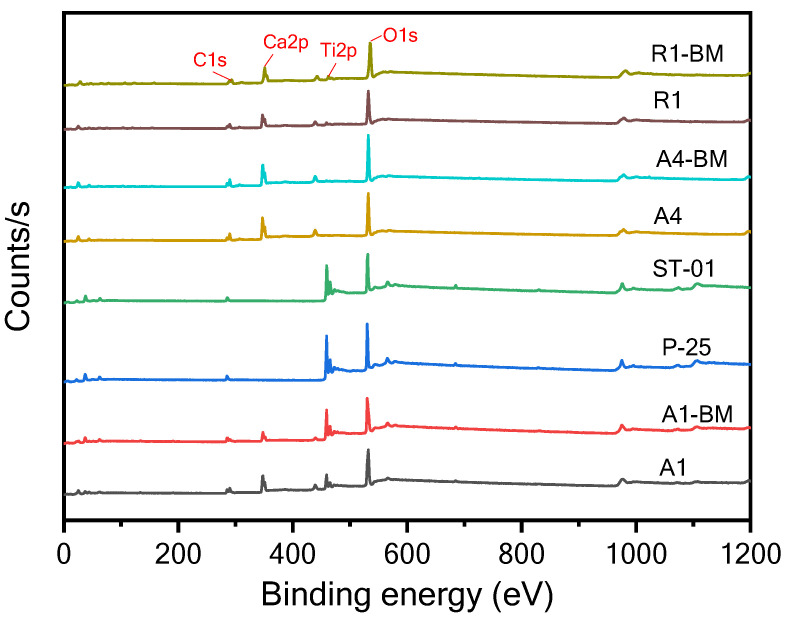
Survey level spectra of the photocatalysts.

XPS is used to obtain qualitative/quantitative information in terms of the chemical composition and electronic structure of the powder samples in Table 1. The survey level XPS analysis of the various TiO_2_ samples (Figure 10) provides the elemental composition (Table S5) of the samples listed in Table 1.

On perusal of Table S5, one can notice that P-25 and STO1 are comprised of elements such as Ti, O, and C, whilst A1, A1-BM, A4, A4-BM, R1 and R1-BM samples additionally have the element Ca. The element C in P-25 and STO1 samples was mainly ascribed to adventitious hydrocarbon from XPS itself. However, the elemental C is present in other samples and can also be ascribed to the presence of CaCO_3_. The Ca elemental % in the samples A1, A4 and R1 are 12.05, 15.81 and 11.44, respectively. It must be noted that A1 contains a large proportion of CaCO3 in the sample (Table 1) as compared to the A4 and R1 samples. However, the composition trend is not reflected in the Ca elemental % in the A1, A4 and R1 samples (Table 1). This is because XPS is a confined surface analysis technique and hence the elemental Ca in A1, A4 and R1 through XPS represents the surface composition of the samples. The Ca elemental % of the A1, A4 and R1 samples show a significant decrease upon BM treatment. Particularly, the Ca elemental % of A1 largely decreased upon BM treatment. The decrease in Ca elemental surface composition can be attributed to two possible reasons. Firstly, BM treatment can cause changes in particle sizes of both TiO_2_ and CaCO_3_ particles as well particle mixing. Secondly, there can be inclusion of Ca ions in the interstitial structure of TiO_2_. 

#### 3.6.2. Core Level Analysis

**Figure 11:**
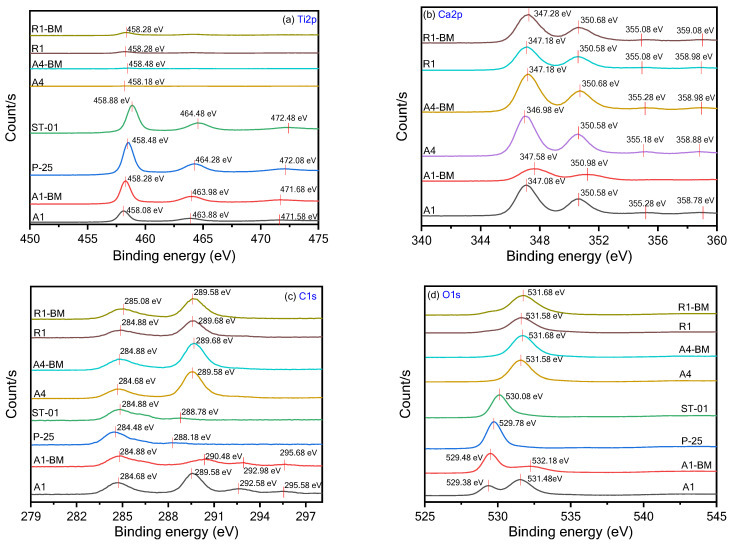
XPS core-level spectra of the photocatalysts in BE intervals of (**a**) Ti2p, (**b**) Ca2p, (**c**) C1s and (**d**) O1s.

Quantitative XPS analysis was performed on the TiO_2_ samples to infer the electronic states of the elements Ti, Ca, O, and C. The high-resolution spectra of the samples are presented in Figure 11a–d.

Upon analyzing Ti 2p states in various samples (Figure 11a), the Ti 2p_1/2_ and Ti 2p_3/2_ spin-orbital splitting photoelectrons for the P-25 and STO1 samples are located at binding energies (BE) of 464.28 and 458.48 eV, 464.48 and 458.08 eV, respectively, indicating the presence of the Ti^4+^ oxidation state [31]. The separation of BE between Ti 2p_1/2_ and Ti 2p_3/2_ states was found to be 5.80 eV and 5.60 eV between the Ti 2p_1/2_ and Ti 2p_3/2_ signals, which is in agreement with the reported literature values [32]. A satellite peak was also observed at 472.08 eV and 472.48 eV for P-25 and STO1, respectively [33,34]. For the A1 sample, the BE for the Ti 2p_1/2_ and Ti 2p_3/2_ states was found to be 463.88 eV and 458.08 eV, respectively. For the A1-BM sample, the BE of the 2p_1/2_ and Ti 2p_3/2_ states was shifted to higher BE levels from the pristine A1 samples with BE values of 463.98 eV and 458.28eV, respectively. The shifting to higher BEs 2p_1/2_ and Ti 2p_3/2_ states for A1-BM sample suggests a decrease in the electron charge density of the Ti^4+^ ion and this could possibly be due to the columbic or chemical interactions between TiO_2_ and CaCO_3_ particles. It must also be noted that there is a shift in higher BE for the satellite peak, also between the A1 and A1-BM sample, implying the possible alteration in electronic state of Ti^4+^ in TiO_2_. Interestingly, the R1 and A4 samples show broad and less intense Ti 2p_1/2_ and Ti 2p_3/2_ BE peaks due to the lower contents of TiO_2_ in those samples (Table 1).

Figure 11b presents the core level XPS spectra of the Ca 2p level in A1, A1-BM, A4, A4-BM, R1 and R1-BM samples. The peaks that correspond to Ca 2p_3/2_ and Ca 2p_1/2_ can be seen at 347.1 and 350.6 eV, 347.6 and 351.0 eV, 347.0 and 350.6 eV, 347.1 and 350.7 eV, 347.3 and 350.7 eV, and 347.2 and 350.6 eV, respectively, which agree with the BEs of Ca in CaCO_3_ reported in the literature [35]. It can be noticed that the Ca BE peaks in the A1 sample are shifted to higher BE values upon BM treatment, whilst no significant shift in BE values was noticed between A4 and R1 samples and their BM treated counterparts. It is inferred that A1 that contains a major proportion of TiO_2_ along with CaCO_3_ is susceptible to chemical interactions between them as compared to the A4 and R1 samples that contain a minor proportion of TiO_2_ in them. 

The C 1s core level spectra are presented in Figure 11c. The C1s spectrum of A1, A4 and R1 comprises of two clearly resolved peaks that are around 284.7 eV (adventitious carbon: C_x_H_y_) and 289.3 eV (carbonate: CO_3_) [36]. These peaks represent different carbon–oxygen bonds in the compounds [37]. Importantly, the high ratio of intensities in carbonate carbon/adventitious carbon means that the A1, A4 and R1 samples have predominantly carbonate carbons but with varying ratio between carbonate carbon/adventitious carbon proportions. There is not much variation in the peak ratios of carbonate carbon/adventitious carbon for A4 and R1 samples informing that BM treatment has negligible effect on the carbonate carbon/adventitious carbon ratio. However, the ratio of C1s carbonate carbon/adventitious carbon peaks drastically decreased between the A1 and A1-BM samples. Importantly, the amount of adventitious carbon may be in dominant proportions for A1-BM as inferred from the ratio. This suggests that the surface of A1 contains a larger proportion of adventitious carbon. On perusal of Table S5, it can be seen that elemental C % increased for A1 upon BM treatment. The XPS results from previous researchers also suggest the presence of adventitious carbon in titania [38,39]. Interestingly, based on computational analysis, it is inferred that the presence of carbon in the surface of titania can hasten the interaction of Ti ions with the other elemental components [40]. Adventitious carbon has also been reported to participate in the enhancement of photocatalytic activities [41,42]. The O 1s core level spectrum of P-25 and STO1 (Figure 11d) consists of an oxide species at BE of 529.4 eV that corresponds to metal oxide bonds, confirming that the sample contains TiO_2_. However, the O 1s core level spectrum of A4, A4-BM, R1 and R1-BM shows a peak around 531.6 eV (Figure 11d) that corresponds to the C=O bond in CaCO_3_. The BE of the O 1s core level peak corresponding to TiO_2_ (529.4 eV) gets slightly shifted by 529.5 eV upon BM treatment for A1. Interestingly, the A1 sample shows an additional O1s peak at 531.5 eV and this peak gets shifted to 532.2 eV. The peaks at 531.5 and 532.2 eV might arise from C-O, or C=O bonds due to adventitious carbon. The deconvolution of core level peaks of Ti, Ca, O, and C elements for the A1+BM sample has been performed and is presented in Figure S4. 

### 3.7. Particle Size Analysis

**Figure 12:**
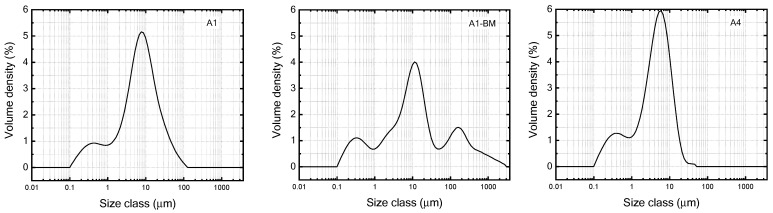

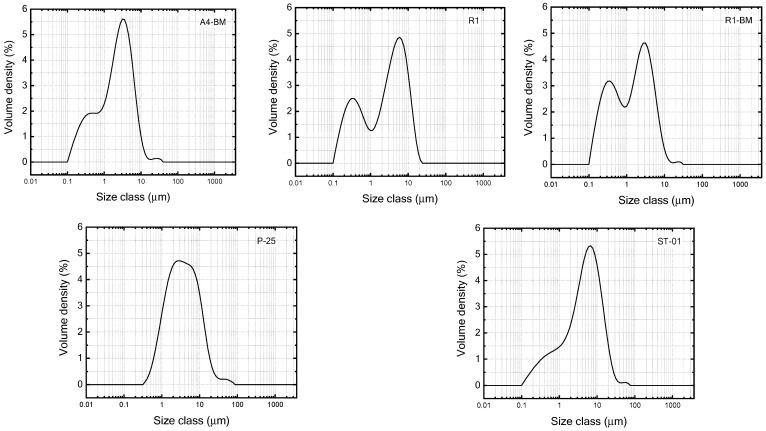
Particle size profiles of the used photocatalysts.

Figure 12 presents the volume density (%) based particle size distribution curves determined by dynamic light scattering (DLS) for various photocatalysts (listed in Table 1 as well their BM treated ones). While ST01 and P-25 show unimodal narrow distributions, the other samples present bi or multimodal size distributions inferring the probable presence of various kinds of physical/chemical particles. The values for D [3,2], D [4,3], specific surface area (S.S.A), d10, d50 and d90 are listed in Table S6.

## 4. Conclusions

This work demonstrates a simple and cost-effective strategy involving the ball milling post treatment of commercially cheaper TiO_2_ (TiO_2_; titania) to enable the use of TiO_2_ on a large scale in the construction industry. The following conclusion can be drawn:

- This work proved that besides the use of pristine pure commercial TiO_2_, cheaper TiO_2_ composite can be suitably modified and incorporated into cementitious materials to develop newer and advanced construction materials.
- Our ball milling post-treatment strategy of composite TiO_2_ demonstrated a significant enhancement in functional performance such as photodegradation capability. The developed construction materials in this work exhibited superior performance over pristine titania due to the augmented interactions between the titania and the component in the composite, modification of crystal structure, surface characteristics and physicochemical properties resulting in influences on the photocatalytic performance of a cementitious system.
- The findings of the present work are expected to contribute to new directions for developing cheaper commercial construction materials with enhanced performance and provides scope for extending the possible utilization of such developed materials in the wider use of photocatalytic building materials.

## Figures and Tables

**Table 1:** Properties of the photocatalyst samples used.

Sample ID.	Company Names	Composition			Price (won/kg)
		A (%)	R (%)	C (%)	
A1(BA01-01)	Hengyang Yutu Chemical (Hengyang, China)	42.6	-	57.4	1000
A4(BA01-01+)	Hengyang Yutu Chemical (Hengyang, China)	-	0.1	99.9	1200
R1(R996)	Sichuan Lomon Group (Sichuan, China)	-	minor	main	2800
P-25	Degussa Ag (Marl, Germany)	87.5	12.5	-	130,000
ST-01	Ishihara Sangyo (Osaka, Japan)	100	-	-	140,000

A, R, and C denote anatase, rutile, CaCO_3_, respectively.

**Table 2:** Mortar mix proportions.

Mixture Types	Water (g)	Cement (g)	Sand (g)	Photocatalysts (%)
Plain mortar	60	100	300	0
Mortar mixed with photocatalysts	60	100	300	5
	60	100	300	10
	60	100	300	15

**Table 3:** Photocatalytic MB degradation efficiencies of various samples in Table 1.

Irradiation Time (h)	Photocatalysts							
	A1	A1-BM	A4	A4-BM	R1	R1-BM	P25	ST01
	Concentration of the Photodegraded Methylene Blue (mg/L)							
1 h	5	3	9.3	8.1	9.3	8.5	3.74	6.31
2 h	1.8	0.67	8.7	7	8.8	7.6	0	3.9

**Table 4:** Pseudo-first order rate constants of the photocatalysts used.

Sample ID.	Pseudo-First Order Rate Constant (Y)	Slope of Y	Regression Coefficient (R^2^)
A1	−0.893x + 0.092	−0.893	0.985
A1-BM	−1.385x + 0.133	−1.385	0.991
A4	−0.066x + 0.001	−0.066	0.990
A4-BM	−0.174x − 0.015	−0.174	0.992
R1	−0.071x − 0.0002	−0.071	0.984
R1-BM	−0.137x − 0.015	−0.137	0.981
P-25	−1.059x + 0.040	−1.059	0.996
ST-01	−0.441x − 0.027	−0.441	0.982

**Table 5:** Summary of the results of the XRD analysis of TiO_2_ and BM treated photocatalysts.

Samples	Composition			2θ			Crystallite Size (nm)			D-Spacing (Å)			Lattice Constants (a, b, c)					
	A (%)	R (%)	C (%)	A	R	C	A	R	C	A	R	C	A		R		C	
				(101)	(110)	(104)							a = b	c	a = b	c	a= b	c
A1-BM	33.3	-	66.7	25.34	-	29.46	48.46	-	42.12	3.51		3.02	3.77	9.50	-	-	4.97	17.02
A1	42.6	-	57.4	25.38	-	29.50	51.21	-	43.01	3.50		3.02	3.77	9.45	-	-	4.97	16.98
A4	-	0.1	99.9	-	-	29.45	-	-	54.39	-	-	3.03	-	-	-	-	4.98	17.02
A4-BM		1.9	98.1	-	27.42	29.38	-	68.9	39.36	-	3.24	3.03	-	-	5.59	2.96	4.99	17.05
R1	-	minor	main	-	27.86	29.46	-	38.6	42.34	-	3.19	3.02	-	-	4.52	2.98	4.98	17.01
R1-BM	0.2	13.2	86.7	25.40	27.46	29.47	67.00	57.35	44.70	3.50	3.24	3.02	3.78	9.51	4.58	2.95	4.97	17.01
P-25	87.5	12.5	-	25.32	27.44	-	18.89	28.79	-	3.51	3.24	-	3.78	9.50	4.59	2.95	-	-
ST01	100	-	-	25.29	-	-	6.28	-	-	3.51	-	-	3.78	9.50	-	-	-	-

A, R, and C denote anatase, rutile, and CaCO_3_, respectively.

## Data Availability

Data are contained within the article.

## Supplementary Materials

The following supporting information can be downloaded at: https://www.mdpi.com/article/10.3390/ma16144931/s1.
