# Supplementary material for: Facile and Simple Post Treatment Ball Milling Strategy for the Production of Low-Cost TiO2 Composites with Enhanced Photocatalytic Performance and Applicability to Construction Materials
Source: Materials (Basel). 2023 Jul 10;16(14):4931. doi: 10.3390/ma16144931 (PMC10381376; doi:10.3390/ma16144931)
Supplement: Supplementary file 1 [file materials-16-04931-s001.zip › materials-2446476-supplementary.pdf]

materials

Supplementary Materials

Facile and Simple Post Treatment Ball Milling Strategy for the Production of Low-Cost TiO_2 Composites with Enhanced Photocatalytic Performances and Applicability to Construction Materials

Supporting Information- Characterization Details (SI-CD)

Experiments	Instruments	Manufacturer
XRD	Multi-purpose X-ray Diffractometer	Malvern PANalytical
XPS	The K-alpha X-ray photoelectron spectrometer	ThermoScientific
DRS	diffuse reflector	SCINCO CO., Ltd., Korea
SEM	SU8220	Hitachi High-Technologies Corporation, Japan
PSA	Mastersizer 3000	Malvern PANalytical
Ball Milling	AV-1 ball miller	AS ONE Corporation in Japan

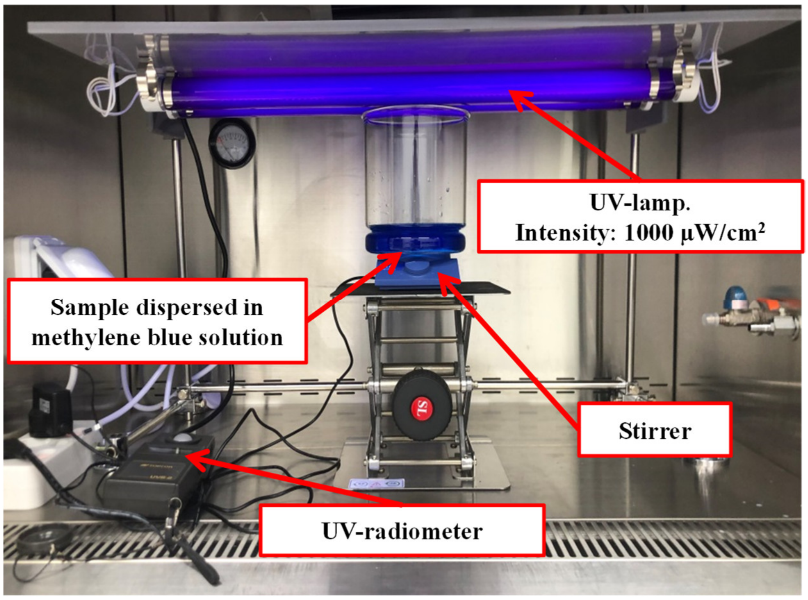

Figure S1. Configuration of methylene blue photodegradation experimental setup.

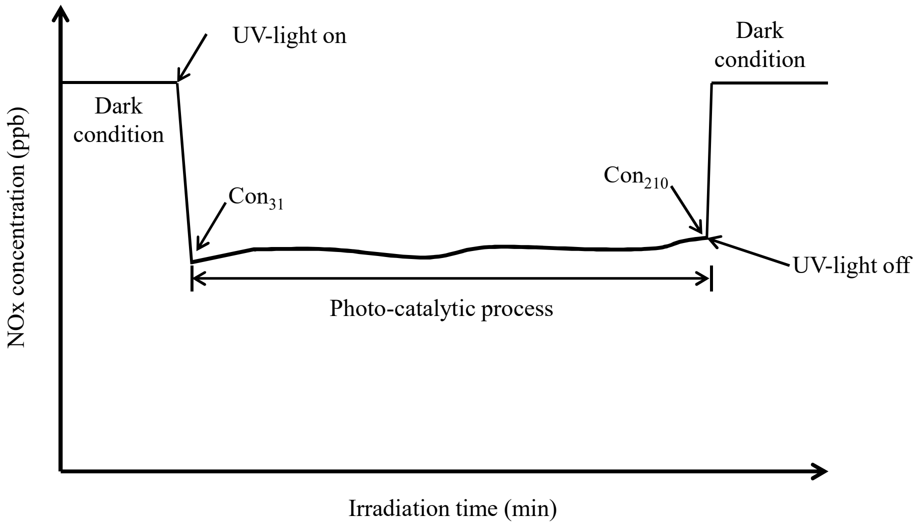

Figure S2. Typical photocatalytic NOx removal measurement scheme.

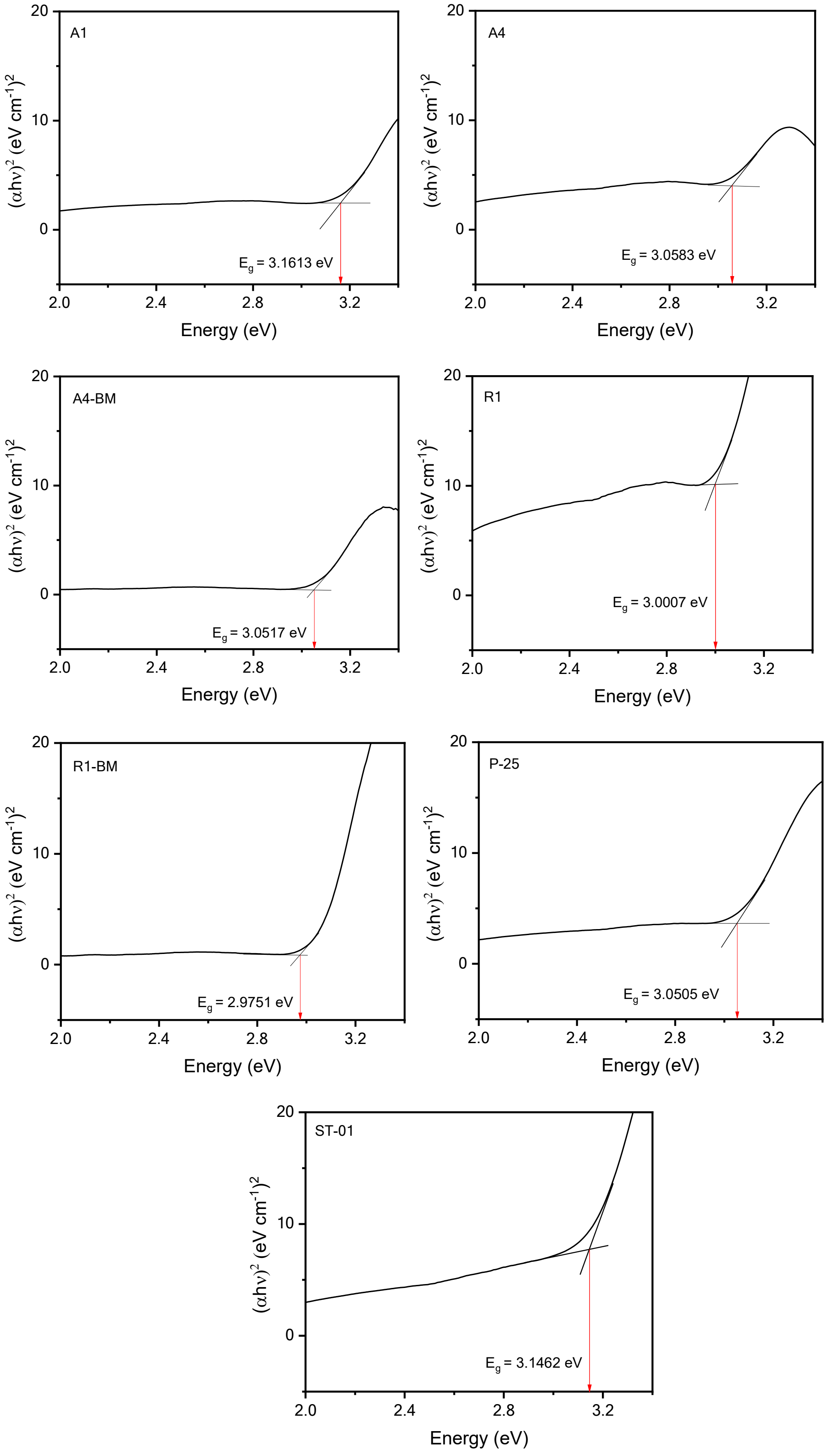

Figure S3. Tauc plots of the photocatalysts used.

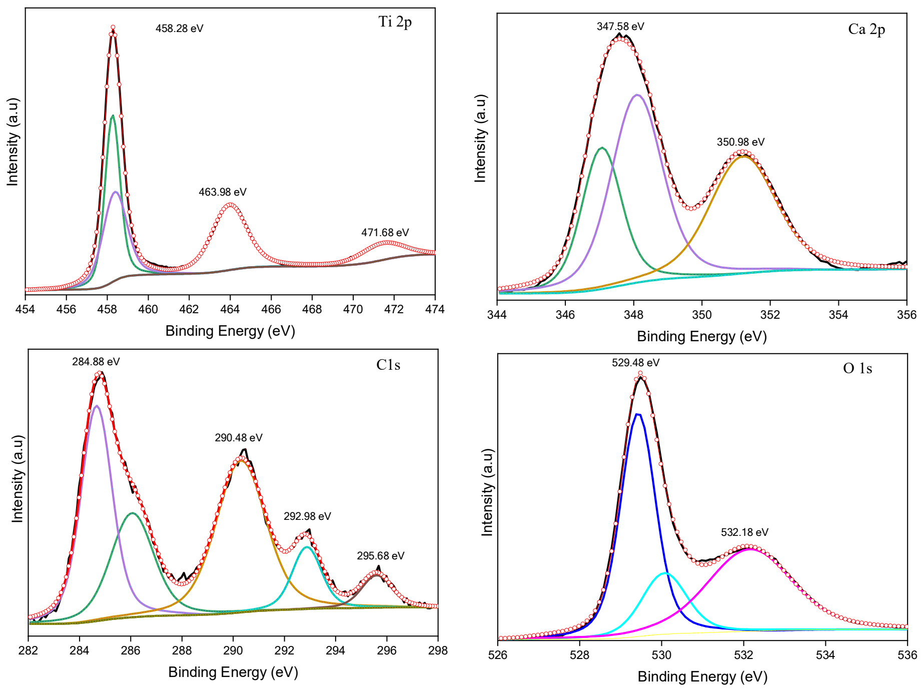

Figure S4. The XPS spectra of A1-BM photocatalyst.

Table S1. NOx photodegradation efficiency of mortar plate coated with the photocatalysts.

Irradiation time (h)	Photocatalysts							
	**A1**	**A1-BM**	**A4**	**A4-BM**	**R1**	**R1-BM**	**P25**	**ST01**
	NOx photodegradation efficiency (%)							
2h	42.6	57.9	3.6	4	6.5	7.6	85	54
3h	43	58	3.9	4.5	7	8	85.9	54.2

Table S2. NOx photodegradation efficiency of mortar mixed with the photocatalysts.

Irradiation time (h)	Photocatalysts				
	**A1-BM 5%**	**A1-BM 10%**	**A1-BM 15%**	**P25 15%**	**ST01 15%**
	NOx photodegradation efficiency (%)				
2h	18.8	25.4	27.6	60	43
3h	19	26	28	60.4	44.6

Table S3. Summary of the band gap energy of TiO_2 photocatalysts.

TiO_2 photocatalysts	Band bag energy (eV)
A1	3.1613
A4	3.0583
R1	3.0007
P-25	3.0505
ST-01	3.1462
A1-BM	3.1563
A4-BM	3.0517
R1-BM	2.9751

Table S4. Summary of particle size of the photocatalysts obtained from SEM images.

A1	A1-BM	A4	A4-BM	R1	R1-BM	P25	ST01
Particle size (nm)							
196.7	36.3	88.5	59	95.1	37.7	13.1	80.3
98.1	180.3	55.7	32.7	336.1	26.2	9.8	60.6
133.5	131.1	245.9	236	295	322.9	32.7	6.5
90.1	16.4	44.2	31.1	32.7	186.8	27.8	45.8
136	18	54.1	96.7	73.7	195	86.8	24.5
Particle size average (nm)							
130.9	76.4	97.7	91.1	166.5	153.7	34	43.5

Table S5. Summary of atomic percent.

Name	Photocatalysts							
	A1	A1-BM	A4	A4-BM	R1	R1-BM	P25	ST01
	Atomic (%)							
C1s Scan A	6.47	5.49	-	-	-	-	-	-
C1s Scan B	11.98	6.22	-	-	-	-	-	-
C1s Scan C	2.1	4.02	-	-	-	-	-	-
Ca2p	12.05	6.56	15.81	14.76	11.44	10.87	-	-
Ti2p	8.75	18.02	0.38	0.64	2.2	2.81	28.7	27.36
O1s	54.11	54.99	56.13	56.25	58.1	58.31	55.19	54.96
C1s	-	-	25.55	25.12	22.15	20.5	12.19	12.45

Table S6. Summary of particle size analysis results deduced from dynamic light scattering.

Samples	Conc[1] (%)	Span	U[2]	S.S.A[3] (m^2/kg)	D[3,2] (μm)	D[4,3] (μm)	D(10) (μm)	D(50) (μm)	D(90) (μm)
A1	0.0008	3.614	1.154	3016	1.99	11.9	0.718	7.44	27.6
A1-BM	0.0006	21.638	8.323	3279	1.83	101	0.528	11.6	25.3
A4	0.0005	2.536	0.801	4146	1.45	5.76	0.477	4.61	12.2
A4-BM	0.0004	2.609	0.877	6036	0.994	3.20	0.347	2.43	6.70
R1	0.0003	3.106	1.004	6943	0.864	4.33	0.274	3.24	10.3
R1-BM	0.0005	3.343	1.130	8890	0.675	2.40	0.245	1.60	5.59
KA-100	0.0003	4.068	1.626	7081	0.847	10.6	0.261	5.73	23.6
P-25	0.0003	3.067	1.073	2393	2.51	5.94	1.11	3.71	12.5
ST-01	0.0008	2.810	0.905	3552	1.69	6.84	0.621	5.09	14.9

[1] Concentration. [2] Uniformity. [3] Specific surface area.
